# Evolution of breeding plumages in birds: A multiple‐step pathway to seasonal dichromatism in New World warblers (Aves: Parulidae)

**DOI:** 10.1002/ece3.6606

**Published:** 2020-08-09

**Authors:** Ryan S. Terrill, Glenn F. Seeholzer, Jared D. Wolfe

**Affiliations:** ^1^ Museum of Natural Science Louisiana State University Baton Rouge Louisiana USA; ^2^ Moore Lab of Zoology Occidental College Los Angeles California USA; ^3^ Department of Ornithology American Museum of Natural History New York New York USA; ^4^ School of Forest Resources and Environmental Science Michigan Technological University Houghton Michigan USA

**Keywords:** feathers, phylogenetic comparative methods, selection, trait evolution

## Abstract

Many species of birds show distinctive seasonal breeding and nonbreeding plumages. A number of hypotheses have been proposed for the evolution of this seasonal dichromatism, specifically related to the idea that birds may experience variable levels of sexual selection relative to natural selection throughout the year. However, these hypotheses have not addressed the selective forces that have shaped molt, the underlying mechanism of plumage change. Here, we examined relationships between life‐history variation, the evolution of a seasonal molt, and seasonal plumage dichromatism in the New World warblers (Aves: Parulidae), a family with a remarkable diversity of plumage, molt, and life‐history strategies. We used phylogenetic comparative methods and path analysis to understand how and why distinctive breeding and nonbreeding plumages evolve in this family. We found that color change alone poorly explains the evolution of patterns of biannual molt evolution in warblers. Instead, molt evolution is better explained by a combination of other life‐history factors, especially migration distance and foraging stratum. We found that the evolution of biannual molt and seasonal dichromatism is decoupled, with a biannual molt appearing earlier on the tree, more dispersed across taxa and body regions, and correlating with separate life‐history factors than seasonal dichromatism. This result helps explain the apparent paradox of birds that molt biannually but show breeding plumages that are identical to the nonbreeding plumage. We find support for a two‐step process for the evolution of distinctive breeding and nonbreeding plumages: That prealternate molt evolves primarily under selection for feather renewal, with seasonal color change sometimes following later. These results reveal how life‐history strategies and a birds' environment act upon multiple and separate feather functions to drive the evolution of feather replacement patterns and bird coloration.

## Graphical abstract

We investigate the evolution of a biannual molt strategy in the New World warblers and find that natural selection likely contributes more to molt evolution than interannual coloration change.

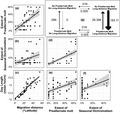

## 1 INTRODUCTION

When subject to dissimilar selective forces, traits that arose for one function often diversify to serve another (Barve & Wagner, 2013). Bird feathers are as diverse in purpose as they are in form, reflecting repeated evolution of novel functions since their origin in early Archosauria (Dimond, Cabin, & Brooks, 2011; Seebacher, 2003). The array of feather functions in birds is the product of separate, and potentially competing, selective forces that have influenced the evolution of feather structure and color over time (Dunn, Armenta, & Whittingham, 2015). Broadly, feather diversity is shaped by natural selection imposed by environmental conditions and by social selection (Dale, Dey, Delhey, Kempenaers, & Valcu, 2015; Lyon & Montgomerie, 2012). Selection often produces bright or gaudy plumages in response to social competition (Karubian, 2002; Rubenstein & Lovette, 2009; SætreDale & Slagsvold, 1994; West‐Eberhard, 1979), while other selective forces on feathers may enhance structural integrity for functions such as flight and thermoregulation; or produce cryptic plumages to help birds hide from their predators and prey. Selective forces vary throughout a birds' annual cycle, and this variability has been hypothesized to lead to the distinctive breeding and nonbreeding plumages shown by many species, that is, seasonal dichromatism (Mulder & Magrath, 1994). Plumage color change in birds has long interested researchers (Beltran, Burns, & Breed, 2018; Chadbourne, 1897; Holmgren & Hedenström, 1995; McQueen et al., 2019; Simpson, Johnson, & Murphy, 2015; Tökölyi, Bókony, & Barta, 2008), but much remains to be discovered about the selective forces that shaped seasonal changes in avian plumage coloration.

Feathers are lightweight, and in order to maintain feather function, all birds replace their feathers at least once per year through molt. Without well‐timed molts, birds can quickly lose functions of feathers such as thermoregulation and flight. Seasonal dichromatism is commonly acquired through biannual molts that produce plumages with disparate phenotypes. While much study has focused on evolution of structure and color in feathers (Dale et al., 2015; Prum, 2005), our understanding of the selective forces and evolutionary pathways which gave rise to disparate molt patterns and strategies remains poor. The annual, complete molt all birds undergo is termed the *prebasic* molt and generates the *basic* plumage. In addition to the prebasic molt, many species of birds undergo a second molt within their annual cycle, termed the *prealternate* molt, which generates the *alternate* plumage and typically corresponds to what is colloquially known as the breeding plumage (Wolfe, Johnson, & Terrill, 2014). The prealternate molt varies broadly in presence and extent among taxa, as well as the amount of phenotypic change it produces. Many species of birds have alternate plumages that are identical to their basic plumages, while others exhibit markedly different alternate and basic plumages. Some species show plumages that are so different that basic and alternate plumaged birds of the same species were originally described as separate species, for example, Black‐bellied Plover (*Pluvialis squatarola*; Poole, Pyle, Patten, & Paulson, 2016). Different species of birds exhibit diverse molt strategies across the globe (Stresemann & Stresemann, 1966). What factors have influenced the evolution of divergent molt strategies? When feathers are replaced more than once a year, is this in response to reduced quality of worn feathers, or to grow feathers with a new phenotype?

Two hypotheses exist to explain the evolution of seasonal dichromatism in birds. The first hypothesis, which we term the *variable pressures hypothesis*, concentrates on feather color and states that prealternate molt evolved in response to differential relative levels of social and natural selection throughout the year (McQueen et al., 2019; Simpson et al., 2015; Tökölyi et al., 2008). This hypothesis is based on the observation that social selection for bright plumage is stronger during the breeding season (Butcher & Rohwer, 1989; Hill, 1991; Karubian, 2002) and may be weaker outside the breeding season such that natural selection would favor a more cryptic plumage in order to evade detection by predators and prey (Götmark, Post, Olsson, Himmelmann, & Gotmark, 1997; Slagsvold, Dale, & Kruszewicz, 1995). Long‐distance migrant birds experience a brief period of intense sexual selection during the breeding season, which is likely reduced on the nonbreeding grounds, though male–male competition may play a strong role in winter plumages in at least some species (Reudink, Studds, Marra, Kurt Kyser, & Ratcliffe, 2009). There is evidence that this has likely led to a latitudinal gradient in sexual dichromatism in the New World warblers and orioles (Friedman, Hofmann, Kondo, & Omland, 2009; Hamilton, 1961; Simpson et al., 2015). On the other hand, resident species may form pair bonds all year and experience more stable relative levels of sexual and nonsexual selection on feather color throughout the year. Under this hypothesis, the prealternate molt evolved similarly to sexual dichromatism—for plumage color. This hypothesis states that prealternate molt evolves in response to variable pressures on feather colors induced by changes in the relative strength of sexual and natural selection on feathers throughout a birds' annual cycle.

**FIGURE 1:**
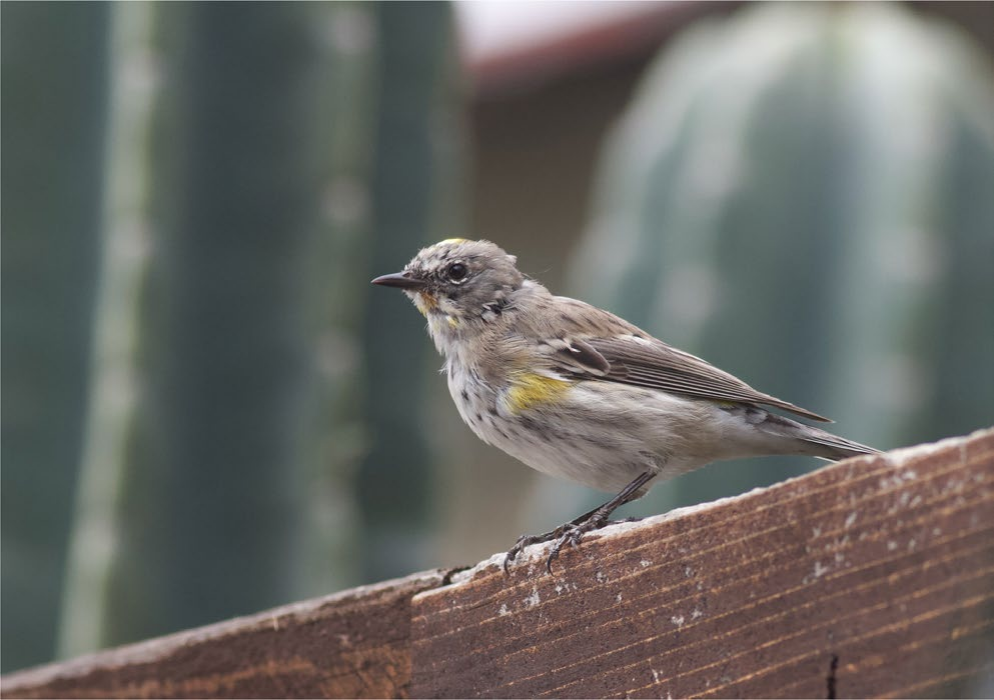
A Yellow‐rumped Warbler (Setophaga coronata) in prealternate molt in Los Angeles, CA. Many birds molt their feathers twice a year, but how and why do these breeding plumages evolve? Photograph: Ryan S. Terrill

**FIGURE 2:**
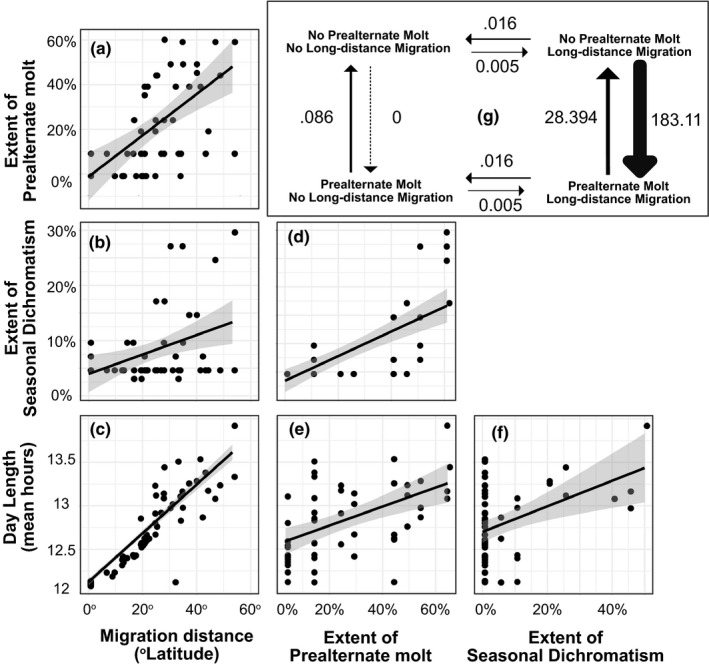
Potential drivers of seasonal dichromatism. (a) The extent of prealternate molt is positively associated with migration distance (pgls: adjusted *R* ^2^ = .19, *F*_1,46_ = 11.79 *p* = .0013), which is also related, to a lesser extent, to (b) extent of seasonal dichromatism (pgls: adjusted *R* ^2^ = .074, *F*_1,46_ = 4.792 *p* = .034). This relationship has led to the hypothesis that migration distance may influence the evolution of prealternate molt. (c) Day length experienced by birds is strongly correlated with migration distance, indicating that long‐distance migrants experience longer days over the year than resident birds (pgls: adjusted *R* ^2^ = .512, *F*_1,46_ = 50.37 *p* < .001). When compared to extent of seasonal dichromatism, prealternate molt shows a positive relationship, but (d) prealternate molt is more extensive on the body than seasonal dichromatism. Day length is also with (e) extent of prealternate molt (pgls: adjusted *R* ^2^ = .16 *F*_1,46_ = 9.974 *p* = .002) and (f) extent of seasonal dichromatism (pgls: adjusted *R* ^2^ = .062, *F*_1,46_ = 4.113 *p* < .048). The low slope of the relationship between extents of prealternate molt and seasonal dichromatism means that warblers generally undergo a prealternate molt that is more extensive than their seasonal phenotype change, that is, much of prealternate molt in the Parulidae does not produce phenotype change. (g) Transition rates estimated under a model of evolution where prealternate molt is dependent on long‐distance migration (favored over independent, AICdep = 194.84, AICind = 255.13, *p* > .001), for gains and losses of prealternate molt and long‐distance migration. We find gains and losses of both traits; prealternate molt is gained at a high rate in species with long‐distance migration, but not in species without long‐distance migration

The second hypothesis, which we term the *feather wear hypothesis*, is focused on feather structure. It is based on an observation that prealternate molts appear to be more common in long‐distance migrants than in nonmigratory species and does not always produce plumage color change (Figure 2). Pyle and Kayhart (2010) and Wolfe (2011) observed that a prealternate molt that produces feathers with the same coloration as prebasic molt is a widespread phenomenon in birds and proposed that prealternate molt may not evolve for breeding plumage necessarily. Instead, they proposed that prealternate molt evolves to replace worn feathers and then can be co‐opted by pressures for seasonal dichromatism. The idea that the realization of selection on plumage color is limited by preexisting molts is not entirely novel. Rohwer and Butcher (1988) investigated delayed plumage maturation in birds and found that molt limitations explained patterns of plumage color better than explanations based on social selection alone. The *feather wear hypothesis* similarly views feather color development through the lens of molt limitations and proposes that the relationship between long‐distance migration and prealternate molt may be associated with the need to replace feathers worn by ultraviolet radiation, where migration degrades feathers through extended photoperiods experienced throughout the year (Lennox & Rowlands, 1969; Surmacki, 2008). This idea is supported by theoretical models demonstrating that biannual molt should evolve when poor feather quality has elevated impacts on survival rates (Holmgren & Hedenström, 1995). Migrant breeders experience longer days and increased feather wear through bleaching during their summer breeding seasons at temperate latitudes relative to resident tropical species (Figure 1c). Thus, the *feather wear hypothesis* is that prealternate molt evolved to replace worn feathers associated with a migratory lifestyle and increased solar exposure during longer days, and then functioned as mechanistic platform for the evolution of seasonal dichromatism following the *variable pressures hypothesis*. The *feather wear hypothesis* does not rule out variable pressures on feather colors, but instead proposes a different mechanism for the origin of prealternate molt. The feather wear hypothesis is a multiple‐step evolutionary process for the evolution of seasonal dichromatism: Prealternate molt evolved to replace feathers and was subsequently co‐opted for seasonal dichromatism in response to differential selective forces at different times of year.

We examined these two hypotheses using the ecologically diverse New World warbler (Parulidae) family, which exhibit remarkable variation in plumage characteristics and migratory behaviors. Variation in molt strategies in this family is accompanied by gains and losses in migratory behavior (Winger, Lovette, & Winkler, 2011) as well as considerable variation in life‐history characteristics, making them a suitable taxonomic group to assess how interactions between separate selective forces influenced the evolution of seasonal dichromatism. To test these hypotheses, we implemented a phylogenetic comparative approach and quantified the extent of prealternate molt and seasonal dichromatism in the New World warblers, as well as 31 life‐history and environmental characteristics that may affect the evolution of prealternate molts and plumage coloration through natural selection.

## 2 METHODS

### 2.1 Molt and dichromatism scoring

We scored the extent of prealternate molt and plumage dichromatism using a combination of specimen examinations and literature review. All specimens were examined at the LSU Museum of Natural Science. We used a combination of collection date, data from specimen labels, and known molt patterns (Pyle, 1997) to classify individuals by age, sex, and molt stage. Species or life stages not available at the LSUMNS were scored from the literature (Pyle, 1997) or visual examination of published photographs of plumages (Dunn & Garrett, 1997; Stephenson & Whittle, 2013). We defined a dichromatic region as a region with visible color or pattern differences between basic and alternate plumage. We scored dichromatism in feather regions as follows: 1 = region completely dichromatic; 0 = no dichromatism in region; and 0.5 = partial dichromatism or intraspecific variation. In some species, extent of molt and dichromatism differs between the first prealternate molt and definitive prealternate molts. In these cases, we considered only the definitive prealternate molts (Wolfe et al., 2014). We scored molt extent using the same museum and literature resources, through examination of molt limits (Pyle, 1997a). For each body region (Figure 2f), we scored molt as follows: 1 = complete replacement of the feathers in the region; 0 = molt absent from the region; and 0.5 = either partial replacement of the feathers in that region or intraspecific variation in extent of molt.

### 2.2 Life‐history parameters

A birds' lifestyle and environment likely affect selective pressures on the functions of feathers, and so, we attempted to quantify many life‐history and environmental parameters for each species. All calculations of life‐history and environmental parameters were conducted in R version 3.3.1 (R Core Team, 2016). Spatial data were extracted using shapefiles of species distributions provided by Birdlife International and NatureServ (Birdlife International, 2016). We worked with the shapefiles of spatial distributions of Parulidae using the packages GISTools (Brunsdon & Chen, 2014), maptools (Bivand & Lewin‐Koh, 2016), raster (Hijmans, 2016), and geosphere (Hijmans et al., 2005) in R. We chose to quantify life‐history and environmental parameters that reflect factors that may result in feather wear from solar exposure due to distribution and migratory behavior, as well as habitat use and foraging stratum. These parameters are migratory distance, breeding latitude, wintering latitude, day length experienced throughout the year, breeding and winter habitat, breeding and winter foraging stratum, nest type, intensity of solar radiation experienced over the year, intensity of solar radiation experienced in the breeding and wintering range (separately), and precipitation, minimum maximum, and mean temperature, and elevation, on the breeding and wintering ranges, separately. We also calculated body mass. Below, we detail how we measured or scored these parameters.

### 2.3 Migratory distance and latitude

To estimate migratory distance, we divided species into three categories: migrants, which have no spatial overlap between their breeding and nonbreeding distributions; nonmigrants, which have complete overlap between breeding and nonbreeding distributions; and partial migrants, which have some overlap between breeding and nonbreeding distributions. Nonmigrants were always set to zero migratory distance. Using shapefiles of breeding and nonbreeding distribution (Birdlife, 2016), we calculated six separate estimates of migratory distance: 1: distance between the midlatitudes of each distribution; 2 & 3: distance between the maximum and minimum latitudes of each distribution, respectively; 4: distance between maximum latitude of breeding distribution and minimum latitude of nonbreeding distributions; 5: distance between minimum latitude of the breeding distribution and maximum latitude of the nonbreeding distribution; and 6: the great circle distance between the centroids of the points. We used linear models to examine the autocorrelation between these variables and chose the first measure of migratory (distance between midlatitudes) distance to use in further analysis, because it best predicted the other measurements of migration. We calculated the latitude of the breeding and winter ranges of each species as the mean latitude value of each shapefile.

### 2.4 Solar radiation, day length, and climate variables

We calculated solar radiation, day length, temperature, precipitation, and elevation values for each species by extracting spatial data from the distribution shape files used to calculate migratory distance. Data were extracted separately for breeding (May–July) and nonbreeding (November–February) seasons. Although nonbreeding and partial migrant birds may reside in the same location for 12 months, we extracted values from the same periods for all species for consistency. The solar radiation and day length datasets were acquired from the NASA Langley Research Center Atmospheric Science Data Center Surface meteorological and Solar Energy (SSE) web portal supported by the NASA LaRC POWER Project (NASA, 2008). We estimated radiation as the average insolation incident on a horizontal surface per month (hereafter solar radiation) over the course of a year in units of kWh m^−2^ month^−1^. We estimated daylight hours as the average daylight hours a species experiences per month (hr/month). We separated solar radiation into radiation experienced in the breeding and winter ranges separately and combined for an overall average. We also created a new variable to estimate total solar exposure by multiplying solar radiation by day length. We extracted ten climatic variables from WorldClim 1.4 (Hijmans et al., 2005) at 2.5‐min resolution. We extracted breeding and nonbreeding range values from maximum, minimum, and mean temperature, precipitation, and altitude datasets. Temperature is provided and degrees Celsius × 10. To extract solar radiation, day length, and climate variables, we generated 10,000 points randomly within each distribution map polygon. We extracted data from each variable layer at each of the 10,000 points for the breeding and winter months, in the appropriate polygon for each species. We then calculated the mean value for each variable in the breeding and winter distributions.

### 2.5 Habitat and stratum

We created a scoring system for habitat and stratum that roughly estimated solar radiation exposure by species. We scored habitats using the following codes: 0 = tall deciduous forest; 1 = coniferous/montane forest; 2 = riparian/secondary/gallery forest, or broad forest type use; 3 = stunted/young forest; 4 = forest edge; 5 = scrub/marshes; 6 = open habitat. We rated stratum by relative stratum within a habitat using the following codes: 0 = ground or near ground; 1 = understory/undergrowth; 2 = midstory; 3 = subcanopy; 4 = canopy/edge/open. Using data from Dunn and Garrett (1997), Curson (2010), Stephenson and Whittle (2013), Rodewald ^(^ 2015) and Schulenberg (2019), we scored the habitat and foraging stratum during the breeding and wintering periods for each species. We also scored the stratum of nest placement and the nest type from these sources. We coded nest types as the following: 0 = cavity; 1 = dome/closed; 2 = open cup.

## 3 ANALYSIS

We conducted all phylogenetic analyses using a recent, multilocus phylogeny of the Parulidae (Lovette et al., 2010).

### 3.1 Model selection and phylogenetic signal

We fit models of evolution to molt and dichromatism to understand how phylogenetic history and selection may interact with these traits, as well as to inform phylogenetic comparative analyses involving these two traits. To select models of evolution for molts and dichromatism, we fit various models of evolution to the data and phylogeny. We fit models of character evolution using Brownian motion (BM), Ornstein–Uhlenbeck (OU), and early‐burst (EB) (Butler & King, 2004) models in the package geiger in R (Harmon, Weir, Brock, Glor, & Challenger, 2008). We fit models of continuous traits for feather regions and extent of molts and dichromatism, and models of discrete traits for presence of molts and dichromatism. We extracted the sample size‐corrected AIC (AICc) values and parameters from the BM, OU, and EB models for cross‐model comparisons and converted these values to AIC weights to compare models (Revell, 2012). We compared the AICc weights for these three models by calculating AICc weights for each feather tract and for presence and extent of prealternate molt, and seasonal dichromatism. To assess the best model across body regions, we calculated AICc weighted parameter values across feather regions by weighting rate parameters by AICc weights and summed these weighted parameters for molts and dichromatism. We calculated phylogenetic signal as Pagel's lambda in phytools (Revell, 2012) for each molt and sexual and seasonal dichromatism for each body region, as well as presence and extent of molts and dichromatism.

The difference between gains and losses of traits can be important to understand how traits change and interact over evolutionary time. We were interested in knowing when and how often seasonal dichromatism and prealternate molt were gained and lost, and whether these transitions provided insight into the relationship between prealternate molt and seasonal dichromatism. We evaluated the number of transitions and the probability that rates of gains and losses were significantly different for presence of molts and dichromatism by reconstructing ancestral states under equal rates (ER) and all rates different (ARD) models; we compared the log‐likelihoods of each model using a likelihood ratio test to obtain a *p*‐value for rejection of the ER model in favor of the more complex ARD model. This method allowed us to ask whether rates of gains and losses of molts and dichromatism were significantly different from equal. We used a similar test, based on Pagel (1994) to test whether the evolution of prealternate molt is dependent on long‐distance migration, through comparison of likelihood ratios of dependent and independent models of evolution (Figure 1g).

### 3.2 Ancestral state reconstruction and rates of evolution

To understand the evolutionary history of prealternate molt and seasonal dichromatism, among separate species and feather regions, we constructed ancestral state estimates of molts and dichromatism as discrete variables by feather region (Figure 4). We conducted ancestral state reconstruction of presence of molts and dichromatism on the whole body, and by feather region. To convert continuous characters to presence, we converted any nonzero integer to a 1, to indicate that the molt or dichromatism is present in the region of interest. We then evaluated the probability of presence and absence of molts and dichromatism for the entire body and by feather region at each node using a likelihood framework in the package APE (Paradis, Claude, & Strimmer, 2004) in R. We conducted model testing by reconstructing ancestral states under both equal rates (ER) and all rates different (ARD) models and used likelihood ratio tests to choose the best model with which to reconstruct ancestral states to help us understand whether we were correctly evaluating the rates of gains and losses over time. We also evaluated molts and dichromatism as continuous characters, scored as the number of feather regions involved, and reconstructed their ancestral states to evaluate their ancestral states and rates of evolution as continuous characters across the bodies of these birds.

### 3.3 Phylogenetic mixed models for molt and dichromatism extents

We built phylogenetic mixed models to predict the presence and extent of prealternate molt and seasonal dichromatism to understand the relative influences of life‐history and environmental variables on these traits. To build mixed models of exogenous correlates of molt extents, we first conducted pairwise phylogenetic generalized least squares analysis over extents of molt, dichromatism, and exogenous correlates (extended data Table 1) using the package caper (Orme et al., 2013) in R. We examined pairwise PGLS results for strength and significance of interactions and used these interactions to build sets of mixed models to test for the effects of exogenous drivers on extents of molts and dichromatism by examining pairwise interaction between molts, dichromatism, and ecological data, as well as covariation between life‐history and ecological correlates. We evaluated these mixed models using caper, MuMIn (Bartoń, 2016), and Nlme (Pinheiro, Bates, DebRoy, & Sarkar, 2016) in R and organized the models using information theory by ranking models by their AICc score (Table 1).

**TABLE 1:** Phylogenetic controlled linear models predicting the extent of seasonal dichromatism or the difference in feather color between the basic and alternate plumage, and the extent of prealternate molt

Response variable	Model	Adjusted *R* ^2^	*p*	AICc	AIC	AIC weight
Extent of Seasonal Dichromatism	extent of prealternate molt + winter foraging stratum	.39	<.001	115.9	0	0.333
Extent of Seasonal Dichromatism	extent of prealternate molt + breeding foraging stratum	.39	<.001	116	0.1	0.317
Extent of Seasonal Dichromatism	extent of prealternate molt + winter foraging stratum + day length	.39	<.001	117	1.1	0.192
Extent of Seasonal Dichromatism	extent of prealternate molt + winter foraging stratum + breeding foraging stratum	.38	<.001	117.9	2	0.122
Extent of Seasonal Dichromatism	extent of prealternate molt	.31	<.001	120.4	4.5	0.035
Extent of Seasonal Dichromatism	migratory distance + breeding season foraging stratum	.16	.008	131	15.1	0
Extent of Seasonal Dichromatism	migratory distance + winter foraging stratum	.15	.0119	131.8	15.9	0
Extent of Seasonal Dichromatism	migratory distance + breeding foraging stratum + winter foraging stratum	.15	.017	132.7	16.8	0
Extent of Seasonal Dichromatism	winter foraging stratum + breeding average temperature	.12	.0219	133.1	17.2	0
Extent of Seasonal Dichromatism	winter foraging stratum	.08	.0318	134.1	18.2	0
Extent of Seasonal Dichromatism	breeding foraging stratum	.08	.0346	134.3	18.4	0
Extent of Seasonal Dichromatism	migratory distance	.07	.0387	134.5	18.6	0
Extent of Seasonal Dichromatism	day length	.07	.0458	134.8	18.9	0
Extent of Seasonal Dichromatism	breeding minimum temperature	.06	.0489	134.9	19	0
Extent of Seasonal Dichromatism	migratory distance	.06	.0594	135.2	19.3	0
Extent of Prealternate Molt	migratory distance + day length + breeding solar radiation	.28	<.001	173.8	0	0.369
Extent of Prealternate Molt	day length + breeding solar radiation	.22	.0014	175.8	2	0.136
Extent of Prealternate Molt	migratory distance	.19	.0014	176.7	2.9	0.086
Extent of Prealternate Molt	migratory distance + breeding solar radiation	.2	.0025	177	3.2	0.074
Extent of Prealternate Molt	day length + migratory distance	.19	.0037	177.8	4	0.05
Extent of Prealternate Molt	day length + breeding solar radiation + solar radiation	.21	.0043	178	4.2	0.045
Extent of Prealternate Molt	migratory distance + winter solar radiation	.16	.0031	178.3	4.5	0.039
Extent of Prealternate Molt	day length	.17	.0057	178.7	4.9	0.032
Extent of Prealternate Molt	migratory distance + winter solar radiation	.17	.0062	178.9	5.1	0.029
Extent of Prealternate Molt	day length + solar radiation	.17	.0062	178.9	5.1	0.029
Extent of Prealternate Molt	migratory distance + solar radiation	.17	.0064	179	5.2	0.027
Extent of Prealternate Molt	day length + breeding precipitation	.15	.0104	180	6.2	0.017
Extent of Prealternate Molt	day length + winter solar radiation	.2	.0102	180.2	6.4	0.015
Extent of Prealternate Molt	day length + breeding foraging stratum winter foraging stratum + breeding solar radiation	.15	.012	180.3	6.5	0.014
Extent of Prealternate Molt	day length + breeding minimum temperature	.14	.0132	180.5	6.7	0.013
Extent of Prealternate Molt	day length + breeding foraging stratum	.14	.0129	180.5	6.7	0.013
Extent of Prealternate Molt	day length + winter foraging stratum	.12	.0352	182.9	9.1	0.004

Note

Top models for the extent of seasonal dichromatism all include the extent of prealternate molt and foraging stratum, by far the best model for seasonal dichromatism was extent of prealternate molt + breeding foraging stratum. The top models for the prealternate molt include migratory distance, day length, and solar radiation variables. This indicates that prealternate molt likely evolves as a mechanism for the replacement of UV‐damaged feathers.

### 3.4 Phylogenetic ANOVA of drivers of molt and dichromatism in feather regions

Identifying those feather regions being replaced by the prealternate molt can provide clues as to why this molt evolves. To investigate how migratory distance interacts with molts and dichromatism within individual feather regions, we conducted a phylogenetic controlled analysis of variance (ANOVA), for each feather region using the package phytools (Revell, 2012) in R. We investigated the influence of migratory distance on prealternate molt within feather regions by comparing these continuous characters to presence and absence of prealternate molt (Figure 2g). We then conducted Holm's sequential Bonferroni post hoc tests on the phylogenetic ANOVA results to correct for simultaneous test runs.

### 3.5 Phylogenetic path analysis

Because the *feather wear hypothesis* is a multiple‐step hypothesis, it is important to be able to parse direct and indirect relationships between variables. We investigated these direct and indirect relationships using a phylogenetic path analysis, following the method outlined by and Von Hardenberg and Gonzalez‐Voyer (2013) as explained in Garamszegi (2014). Phylogenetic path analysis has several advantages when assessing multivariate relationships, especially in its ability to discriminate between direct and indirect effects between variables, and in its consideration of multiple interactions at once. To evaluate the multivariate interactions in this system, we used results from PGLS analyses to inform 12 separate hypotheses of direct and indirect effects within prealternate molt, seasonal dichromatism, migration distance, and foraging stratum. We used a d‐sep‐based path analysis to build sets of phylogenetic controlled model equations, which we evaluated using the package caper (Orme et al., 2013) in R. We then used an information theory approach based on a C‐statistic (Shipley, 2016) to rank candidate models. The C‐statistic evaluates and ranks the conditional independencies within the models and produces CICc score for each model. We used *p*‐values and CICc (Von Hardenberg & Gonzalez‐Voyer, 2013) scores to evaluate the probability and information content of the C‐statistic, respectively. We used *p*‐values of the C‐statistic to identify a subset of models that we were not able to reject and then ranked models by their CICc score to evaluate the likelihood of each candidate model.

## 4 RESULTS

### 4.1 Ancestral state reconstruction

We found support for an Ornstein–Uhlenbeck (OU) model (AICc weight = 0.96), for presence of prealternate molt, but support for Brownian motion (BM) evolution (AICc weight = 0.60) for extent of prealternate molt. We found support for a BM model for both presence (AICc weight = 0.56) and extent (AICc weight = 0.57) of seasonal dichromatism.

The feather regions more involved in prealternate molt, namely the head, breast, belly, and back, showed higher rates of evolution relative to other feather regions (Figure 4). We reconstructed a partial prealternate molt at the root of the tree, only on the head, with no associated seasonal dichromatism, and several gains and losses of both seasonal dichromatism and prealternate molt (Figure 4), which agrees with our transition analysis (Figure 1) that prealternate molt can be gained and lost over time, over separate lineages (Figure 1e).

### 4.2 Phylogenetic generalized linear models assessing exogenous correlates among extents of molts and dichromatism

For individual pairwise comparisons between variables, we found that extent of seasonal dichromatism was best predicted by extent of prealternate molt (adjusted *R* ^2^ = .312, *p* < .001), day length (adjusted *R* ^2^ = .065, *p* = .046), and migration distance (adjusted *R* ^2^ = .072, *p* = .039), which were correlated with prealternate molt. Seasonal dichromatism was also significantly correlated to foraging stratum (adjusted *R* ^2^ = .078, *p* = .032), which was not correlated with prealternate molt. The extent of prealternate molt was significantly correlated with extent of seasonal dichromatism (adjusted *R* ^2^ = .312, *p* < .001), day length (adjusted *R* ^2^ = .16, *p* = .001), migration distance (adjusted *R* ^2^ = .188, *p* = .013), and breeding latitude (adjusted *R* ^2^ = .109, *p* = .013) (Figure 1).

Sixteen mixed models significantly predicted the extent of prealternate molt with significance of *p* < .05, and we ranked these models using the sample size‐adjusted information theory criterion AIC_c_ (Table 1). The top model for extent of prealternate molt outperformed all other models by a sizable margin, and the top two models combined accounted for the majority of the AIC weight. Top models that predicted the extent of prealternate molt generally included day length, solar radiation both in the breeding and nonbreeding seasons, and migratory distance as predictor variables. In all, we found fifteen models that predicted the extent of seasonal dichromatism with significance of *p* < .05; and these models included the extent of prealternate molt, foraging stratum both in the breeding and nonbreeding seasons, and migratory distance (Table 1). Top models were evaluating seasonal dichromatism more evenly weighted than models for prealternate molt, with the top two models produced similar AIC_c_ values, and the third and fourth models produced similar AIC_c_ values. All four of these top models, which accounted for the majority of the AIC_c_ weight, included extent of prealternate molt and foraging stratum. Foraging stratum, both in the breeding and nonbreeding seasons, was the main predictor variable in the top models for the extent of seasonal dichromatism that was not associated with top models of prealternate molt.

### 4.3 Phylogenetic ANOVA of feather regions

We found that the positive correlation between migratory distance and prealternate molt was repeated across feather regions. In general, migratory distance predicted whether a feather region was replaced during prealternate molt (Figure 2). This relationship was significant in the head (*F* = 13, *p* = .002), breast (*F* = 15.5, *p* = .001), back (*F* = 12.47, *p* = .033), belly (*F* = 14.8, *p* = .013), and tertials (*F* = 11.1, *p* = .015).

### 4.4 Phylogenetic path analysis

Two path models were strongly favored by information theory analyses, with roughly equivalent CICc values. These were models 2 and 3 (Figure 4), both of which proposed that prealternate molt and foraging stratum are direct parent variables of seasonal dichromatism and that migration distance is a direct parent of variable of day length, and only differed in whether migration distance or day length was a direct parent of prealternate molt. The best model that proposed a conditional independency for prealternate molt was model 5, the next best model after models 2 and 3, though this model showed a marked jump in its CICc value compared to models 2 and 3.

## 5 DISCUSSION

### 5.1 The feather wear versus variable pressures hypotheses

The *variable pressures hypothesis*, which proposes that prealternate molt evolves in response to variable selective regimes imposed upon colors of birds' feathers throughout the year, predicts coevolution of prealternate molt and seasonal dichromatism. We found discrepancies in the best‐fit models of evolution, timing, pattern, and external correlates of evolution of between the prealternate molt and seasonal dichromatism. The character that we studied with the strongest evidence for selection, as interpreted by the ratio of likelihood for an Ornstein–Uhlenbeck model to a Brownian motion model, was presence of prealternate molt. This may imply that prealternate molt itself is under stronger selection (Butler & King, 2004) than the coloration it produces, which fit slightly better to a model of Brownian motion. We interpret this as support for the *feather wear hypothesis*, because this hypothesis predicts stronger selection on molt patterns than on seasonal dichromatism. The life‐history characteristics that best predicted prealternate molt were migration distance, day length, and solar radiation experienced on the breeding grounds (Figure 2, Table 1). Top models for seasonal dichromatism all included prealternate molt and foraging stratum on the breeding and nonbreeding ranges. Combined with the results of the path analysis, we interpret these results as evidence for prealternate molt and seasonal dichromatism evolving in separate selective contexts.

The *feather wear* hypothesis invokes preadaptation in the relationship between prealternate molt and seasonal dichromatism in that the prealternate molt may have evolved in response to selective pressures on structural functions of feathers, but then served as a mechanism for response to variable selection on feather colors. The *variable pressures hypothesis* may predict synchronous evolution of prealternate molt and seasonal dichromatism, whereas the *feather wear hypothesis* predicts that prealternate molt should precede seasonal dichromatism and correlate with separate external parameters. When we investigated the evolutionary timing of these characters, prealternate molt appeared to arise before seasonal dichromatism, and in more species and feather regions (Figure 4). The idea that a character can evolve in response to selection for one function, and then be co‐opted to serve another, has been well‐explored in evolutionary biology (Bock, 1959). Preadaptation has been implicated in the evolution of a wide array of evolutionary novelties (Cheney & Seyfarth, 2005; Ketola et al., 2013; Quiñones & Pen, 2017; Schiestl & Cozzolino, 2008) and is an important phenomenon to understand when investigating how traits evolve. Our ancestral state reconstruction suggests that prealternate molt is a preadaptation, rather than an adaptation for seasonal dichromatism. We do not present these results as a rebuttal to variable pressures on feather color. Clearly, functions of feather colors vary with life‐history and latitudinal gradients in social behavior (Friedman et al., 2009), pressure for crypsis on migration induced by predators (Simpson et al., 2015), and nest stratum (Martin & Badyaev, 1996), though we found little support for a relationship between nest stratum or nest type and prealternate molt or seasonal dichromatism. Our findings suggest that latitudinal gradients likely do play a role in the evolution of color change in feathers once prealternate molt is present. From these results, we propose a two‐step pathway for the evolution of disparate breeding and nonbreeding plumages in warblers: A biannual molt evolves in response to structural pressures on feathers and then serves as a preadapted mechanism for seasonal dichromatism.

Feather functions may help explain why structure may influence molt more than color change. Structural functions provided by feathers are more immediately necessary for survival of birds than colors that function for social signaling. Without feathers, chicks are poikilothermic (Whittow & Tazawa, 1991) and reliant on their parents for warmth. In adult birds, worn feathers directly influence survival through decrease of important functions such as flight (Swaddle, Witter, Cuthill, Budden, & McCowen, 1996). Timing of molt appears to be so important that experimentally malnourished birds will undergo a molt in spite of losing up to 40% body mass in the process, instead of delaying molt (Murphy, King, & Lu, 1988). Because of more immediate implications on survival, it may make sense that selection on feather structure is stronger than on color change and that selection on feather structure may be more likely to influence the evolution of molt strategies.

### 5.2 Life‐history and environmental correlates of molts and color change

**FIGURE 3:**
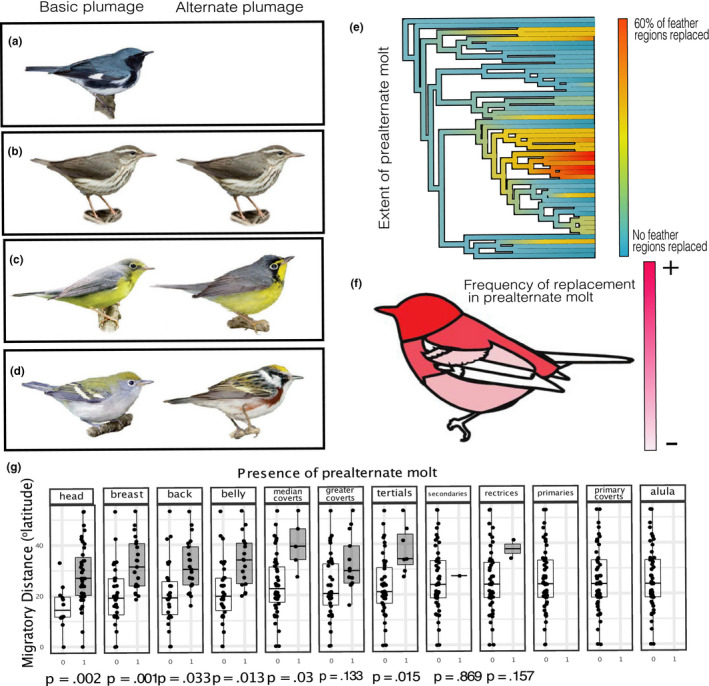
Color change in prealternate molt varies from (a) no prealternate molt to (b) an alternate plumage molt that is identical or nearly so to the basic plumage, or an alternate plumage that is partially (c) or very (d) different from basic plumage. (e) Prelaternate molt has evolved in several lineages, with gains and losses present over the history of the New World warblers. Extent of prealternate molt is strongly (adjusted *R* ^2^ = .19, *p* = .0014) correlated with migratory distance. Blue = no prealternate molt; red = extensive prealternate molt. (f) The frequency of prealternate molt by feather region. Not all warblers undergo prealternate molt, but all that do include the head. There seems to be a stereotyped succession of inclusion of a feather region in prealternate molt as it becomes more extensive in a species, that succession is depicted from left to right from head, to the alula. (g) The relationship between prealternate molt by feather tract and migratory distance. Migratory distance predicts inclusion of a feather region in prealtenate molt in every feather region with *n* > 3 species showing prealternate molt in that feather region

Phenotypic evolution is the result of repeated interactions between selective pressures and preexisting structures available for selection to act upon, in addition to neutral drift. Selection can only work upon biological features that exist, and that contemporary uses for a biological structure may not fully explain why that structure originally evolved. While it may make intuitive sense that prealternate molt is “for” a breeding plumage, and indeed some naming conventions (*e.g.,* prenuptial molt, prebreeding molt) imply this causative relationship, it is important to disentangle direct and indirect causation when attempting to understand how selection interacts with phenotypic evolution over time (Hardenberg & Gonzalez‐Voyer, 2013). Phylogenetic path analysis produced two top models, both of which found that the extent of prealternate molt was associated directly with migratory distance and cumulative annual day length. This suggests that seasonal dichromatism is connected indirectly to migratory distance through prealternate molt (Figure 3b). The models suggested that seasonal dichromatism was determined by the presence of the prealternate molt and foraging stratum, with birds foraging in more open strata experiencing more extensive prealternate molts and seasonal dichromatism. This generally agrees with previous findings that sexual selection operates more strongly in canopy birds, which tend to be more visually oriented (Gomez & Théry, 2004; Shutler & Weatherhead, 1990), resulting in brighter plumages (Shultz & Burns, 2013). From a structural standpoint, canopy birds may also experience greater solar exposure. Indeed, one of the few tropical groups of birds with a known prealternate molt are the becards (*Pachyramphus*; Johnson & Wolfe, 2017) which show identical alternate and basic plumages, and inhabit canopy and forest edge habitats. Importantly, breeding season foraging stratum, when combined with extent of prealternate molt, strongly predicted extent of seasonal dichromatism, but did not by itself predict extent of prealternate molt (Table 1). This suggests that selective pressure on plumage color acts on seasonal dichromatism only after prealternate molt has evolved for other reasons and then provides a structural canvass for sexual selection to paint upon.

Past studies have found that sexual dichromatism can evolve through the loss of a gaudy plumage among female migratory birds (Simpson et al., 2015). Similarly, we find that at least in some cases, year‐round monochromatism evolved through loss of the prealternate molt. It is important to consider phylogenetic context in the evolution of different types of dimorphism because trait gains and losses may mean different things over evolutionary time. For example, Simpson et al. (2015) found sexual dichromatism in warblers stems from loss of bright coloration in females, and Friedman et al. (2009) found a similar pattern in oriole plumage. From the perspective of migratory distance, Winger et al. (2011) showed that resident warblers were more likely to be examples of lineages that had lost long‐distance migration. We found gains and losses of both seasonal dichromatism and prealternate molt, and, importantly, we found that losses of long‐distance migration were associated with loss of prealternate molt. Froehlich, Rohwer, & Stutchbury (2004) and Tökölyi et al. (2008) proposed that the relationship between migratory distance and seasonal dichromatism is caused by earlier breeding in resident species which limited their ability to molt; however, resident species do not appear to be limited in their molts when compared to migrant birds, as they show increased molt–breeding overlap (Johnson, Stouffer, & Bierregaard, 2012) and protracted molts (Kiat, Izhaki, & Sapir, 2019; Terrill, 2018). Furthermore, it is likely that migrant birds are limited in their molt timing, as they generally complete prealternate molt before beginning spring migration (Pyle, 1997b). Without a prealternate molt, nonmigratory warblers are often the same color during the year, and resident warblers fall into two categories, those that are either gaudy all year or cryptic all year. These findings suggest that variable pressures on feather color alone are not strong enough to maintain a biannual molt in these birds, without an external force acting on the structural integrity of their feathers, and long‐distance migration directly impacts structural integrity. Furthermore, selection that affects the latitudinal gradients in sexual dichromatism and seasonal dichromatism likely differs because each is derived from a different mechanism. While sexual dichromatism can be associated with the *prebasic* molt and result in a yearlong plumage aspect, seasonal dichromatism results in discrepancies between the *prebasic* and *prealternate* molt, and results in seasonally variable plumage aspects.

### 5.3 Molt and coloration across feather regions

**FIGURE 4:**
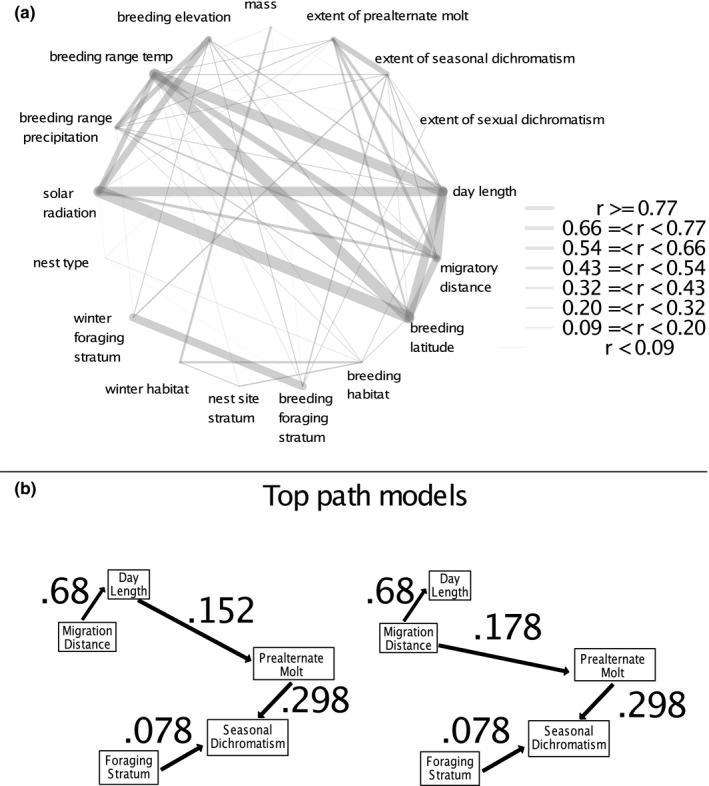
(a) Interactions between variables considered in this analysis, estimated by a phylogenetic controlled linear regression. Width of gray bars indicates *r* values and depicts relative strength of relationships between variables considered. Many strong relationships were expected, such as between temperature and latitude on the breeding grounds, but others, such as between foraging stratum, solar radiation, and migratory distance, help explain extent of both prealternate molt and plumage dichromatism. To investigate multiple‐step interactions, we conducted a phylogenetic path analysis, and the top two models (b) all included migration distance and day length and parent variables to prealternate molt, which is then a parent variable of seasonal dichromatism. We interpret this as evidence, combined with prealternate molts that do not change color aspect, that prealternate molt evolves for the replacement of worn feather and then can be expected for seasonal plumage alteration

**FIGURE 5:**
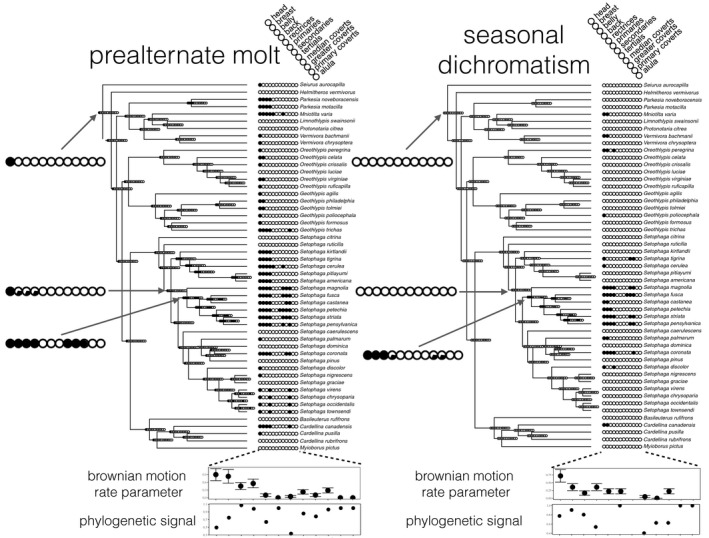
The evolution of prealternate molt and seasonal dichromatism within feather regions in the New World warblers. In all plumage regions, prealternate molt precedes seasonal dichromatism. Both variables are phylogenetically dispersed and follow a predictable pattern, where the head is replaced most often, followed by the breast and belly, down to the alula, which is never replaced. The regions that are replaced more often in prealtenate molt generally show a higher rate of evolution, as measured by the Brownian motion rate parameter. The head shows a low phylogenetic signal, because it is involved in the prealternate molt in many species across the family, while the belly and back show elevated phylogenetic signal, with their presence being clustered into a few clades. Highlighted are three example nodes illustrating how prealternate molt evolves before seasonal dichromatism: first, at the base of the tree, where prealternate molt is reconstructed on the head, with no seasonal dichromatism. Second, at the common ancestor of all *Setophaga*, excluding *S. citrina* and *S. ruticilla*, which do not have a prealternate molt. Here, a shift appears to occur in the tree, where prealternate molt is reconstructed at the head, and with some probability on the breast, belly, and back, though with no accompanying seasonal dichromatism. Third, at the base of a clade of Setophaga with the most extensive prealternate molt and seasonal dichromatism in the family, with PA reconstructed in the head, breast, belly, back, tertials, and median and greater secondary coverts, with all of those tracts reconstructed with some, probability for seasonal dichromatism

Selective pressures on plumage may vary across birds' bodies (Marcondes & Brumfield, 2019; Dale et al., 2015). We know that molt in different species of birds varies in which feathers are molted and when (Stresemann & Stresemann, 1966), but, despite some hypotheses being put forward (Howell, 2010), little work has investigated the interplay between feather function and molt patterns across feather regions. Among species of warblers, certain feather regions were repeatedly more or less likely to be involved in prealternate molt. Despite variation in prealternate molt extent, ancestral state reconstruction suggested that the prealternate molt evolved in a stereotyped manner (Figure 2g; Figure 4). The head is involved in prealternate molt in all species and then most frequently followed by the back, breast, belly feathers, and wing coverts. Prealternate molt rarely replaces other parts of the body, including wing and tail feathers, which are often shaded from the sun by covert feathers and each other. The feather regions more involved in prealternate molt appear to be those more exposed to the sun on a perched bird (Figure 3f). Although the wing and tails are prominent features on birds, when folded, each individual remex is almost entirely shaded by coverts and other remiges (Figure 3). It may also be the case that the larger and stronger remiges are costlier to replace than body feathers, but we interpret this evidence as at least suggestive that feathers that are more exposed to the sun are more likely to be replaced in prealternate molt. This pattern was confirmed by both ANOVA and ancestral state reconstruction, where feather regions most strongly associated with prealternate molt were also correlated with long‐distance migration (Figure 5g) and showed increased rates of evolution in seasonal dichromatism and prealternate molt (Figure 5). In each feather group, we recovered the same positive relationship between migratory distance and likelihood of replacement in prealternate molt (Figure 3g). We found gains and losses of long‐distance migration and prealternate molt and, importantly, found no gains of prealternate molt in birds without long‐distance migration, but a high transition rate to prealternate molt in lineages with long‐distance migration (Figure 3g). The predictable evolution of prealternate molt in regions of the body more exposed to the sun, coupled with a lack of seasonal dichromatism in lineages which recently evolved prealternate molt, lends support to the feather wear hypothesis for the evolution of prealternate molt.

### 5.4 Feather wear and structural function

Photodegradation is a primary source of feather structure atrophy in feathers (Ito, Wakamatsu, & Sarna, 2018; Pearlstein et al., 2014). The main variables that predict extent of prealternate molt are migration distance, day length, and foraging stratum. Migration distance likely affects feather degradation through increased overall day length (Figure 2a). Long‐distance migrants experience longer days overall because they experience long summer days in the temperate zone, but escape short winter days. For example, the longest‐distance migrant in our dataset, and one of the most seasonally dichromatic species with one of the most extensive prealternate molts, the Blackpoll Warbler (*Setophaga striata)* experiences an average of 1.7 more hours of daylight each day, or 621 more hours of ultraviolet exposure each year, when compared to the species exposed to the least amount of ultraviolet radiation, the Masked Yellowthroat (*Geothlypis aequinoctialis*), which also shows no seasonal dichromatism and no prealternate molt. Additionally, many warbler species exhibit prealternate molts that do not result in seasonal dichromatism, and this phenomenon may seem paradoxical from the standpoint of hypotheses focused on coloration as the evolutionary catalyst for the prealternate molt, but makes sense within the context of the *feather wear hypothesis*.

Evidence from other taxa outside the New World warblers provides additional context for the relationship between prealternate molt and seasonal dichromatism. The most extensive prealternate molts in birds occur in three species of long‐distance migrants that breed, winter, and migrate in open, solar‐exposed environments: Franklin's Gull (*Leucophaeus pipixcan*; Howell, 2010 *)*, Bobolink (*Dolichonyx oryzivorus*; Renfrew, Frey, & Klavins, 2011), and Willow Warbler (*Phylloscopus trochilus;* Underhill et al., 1992). Bobolink shows seasonal change in feather color, but Willow Warbler does not, and Franklin's Gull only shows a partial plumage color change. In these species, anecdotally, migration distance and habitat better predict prealternate molt than color change. The Willow Warbler is an extreme example: This species completely replaces all feathers twice a year, but the basic and alternate plumages are indistinguishable. Further research into this phenomenon should expand beyond the New World warblers to other groups of birds, as well as attempt to measure relative feather degradation rates in association with life history, habitat, and environment in birds, and study groups with more variable social systems. Other resident species of birds with strong variable selection on feather color, such as Ptarmigans (Beltran et al., 2018), may indeed have molts that evolve solely for variable pressures on feather color. Overall, our results demonstrate the importance of molt strategies in the functional diversification of feathers and illuminate the value of considering interactions between different functional requirements for birds in the evolution of feather function.

## 6 CONCLUSIONS

Based on the observation that prealternate molt often involves feathers that appear to be identical between basic and alternate plumage, Pyle and Kayhart (2010) proposed that prealternate molt may evolve to replace sun‐exposed feathers, then later be co‐opted for seasonal dichromatism. We examined this hypothesis across warblers and found that it better explains patterns of evolution of prealternate molt than color change alone. Here, we present evidence that selection on coloration and structure interact in complex ways to influence the evolution of molts and plumages in warblers. Namely, we find that color change poorly explains the evolution of the molts that produce these changes. This suggests that biannual molt acts as a preadapted platform for color change, instead of evolving in direct response to variable selective regimes on feather colors. These results provide a more nuanced understanding of plumage evolution in birds by incorporating the mechanism for plumage generation. Rohwer and Butcher (1988) made a novel contribution to our understanding of delayed plumage maturation in birds by arguing that molt must be understood first in order to understand plumage maturation in birds. They found that the breeding season‐driven hypotheses lose support when molt is studied and that the limitations of preexisting molts explain delayed plumage maturation in birds better that social selection on the breeding grounds. Our results largely agree with this study, in that we find variable selective regimes on plumage change do not appear to be able to influence the evolution of molt strategies; instead, they only influence the phenotypes of feathers produced within molt strategies that have evolved for other reasons. Similarly, selective pressures for seasonal color change may be present in species, but the translation of that need into phenotype may be limited by the extent of prealternate molt. Following our results and those of Rohwer and Butcher (1988) that hypotheses about the role of social selection on feather color may look different when viewed through the lens of molt, we encourage other authors studying the evolution of plumage to consider molt strategies when attempting to understand mechanisms of feather evolution.

Feather color has attracted much attention, especially into selective processes that may have produced the diversity of coloration present in birds' feathers (Chaine & Lyon, 2008; Darwin, 1981; Li et al., ; Payne, 1984; Wallace, 1891). Both natural and sexual selection play roles in the colors of feathers as well as diversification of birds (Barraclough, Harvey, & Nee, 1995; Møller & Cuervo, 1998; Stoddard & Prum, 2008; Marcondes & Brumfield, 2019). However, the molts that produce these feathers have been largely ignored in these studies. Birds show a diverse array of molt strategies (Stresemann & Stresemann, 1966), but how and why different species of birds have different strategies for the timing and patterns with which they replace their feathers remains poorly known. How selection interacts with molt, and not just feather phenotype, is an essential question because molt is the underlying mechanism of feather production. How molts provide limitations and opportunities for seasonal change may be of widespread importance for understanding evolution of avian color at a broader scale. For example, some juvenile birds are brightly colored and then lose this bright coloration in the highly conserved preformative molt that occurs shortly after fledging (Pyle, 2009). It may be that the preformative molt provides an opportunity for these chicks to respond to selection from parental choice in the nest (Lyon, Eadie, & Hamilton, 1994) without being “stuck” in a bright plumage for their entire first year of life.

The seasonally differential selective pressures on plumage color likely vary by latitude and social system in birds (Friedman et al., 2009; Simpson et al., 2015), but these results suggest that they may not be the primary factor influencing the evolution of prealternate molt in the New World warblers. A major study into global variation in seasonal plumage coloration in birds found that seasonal color change is more uncommon than predicted by social systems and predation risk (McQueen et al., 2019). We believe our study sheds some light on this conundrum. From the viewpoint of the *feather wear hypothesis*, the answer to this problem is that variable selection on seasonal feather colors is not strong enough to influence molt patterns in many species, and so, seasonal color change can only evolve within the context of preexisting molts. This is similar to how preexisting molts limit the phenotypic realization of plumage maturation (Rohwer & Butcher, 1988). A two‐step relationship between a selective pressure for feather color change and the response of phenotypic evolution to those pressures may not necessarily be unexpected. Selection for color and structure on feathers likely interact in complex ways. For example, sexual selection on feathers may act as a “bridge” between peaks on natural selective landscapes for feather structure (Persons & Currie, 2019). Our results provide evidence for similar “bridges” across adaptive landscapes, where naturally selected molts may provide bridges between spaces on a social selection landscape, in this case between year‐round monochromatism and seasonal dichromatism. Further research into the interplay between different types of selection on the evolution of molts and plumages in birds could consider groups with disparate social systems, as well as quantification of feather degradation. We suggest molt should be considered when attempting to understand the evolution of plumages in birds.

## CONFLICT OF INTEREST

The authors declare no conflicts of interest.

## AUTHOR CONTRIBUTIONS

**Ryan S. Terrill:** Conceptualization (equal); data curation (lead); formal analysis (lead); funding acquisition (equal); investigation (equal); methodology (equal); project administration (lead); resources (lead); software (equal); supervision (lead); validation (equal); visualization (lead); writing – original draft (lead); writing – review and editing (lead). **Glenn F. Seeholzer:** Conceptualization (equal); data curation (supporting); formal analysis (equal); investigation (supporting); methodology (supporting); writing – original draft (supporting); writing – review and editing (supporting). **Jared D. Wolfe:** Conceptualization (equal); data curation (supporting); formal analysis (supporting); investigation (supporting); methodology (supporting); writing – original draft (supporting); writing – review and editing (supporting).

## Data Availability

Data associated with this project are included as supporting information. Code associated with this project is available at https://github.com/enicurus/warbler.molt.migration. Data and code associated with this project have been deposited in Dryad at: https://doi.org/10.5061/dryad.n8pk0p2sm.

## References

[ece36606-bib-0100] Barraclough, T. G. , Harvey, P. H. , & Nee, S. (1995). Sexual selection and taxonomic diversity in passerine birds. Proceedings of the Royal Society of London. Series B: Biological Sciences, 259(1355), 211–215.

[ece36606-bib-0002] Bartoń, K. (2016). MuMIn: Multi‐model inference. R package version 1.15.6. Retrieved from https://CRAN.R‐project.org/package=MuMIn

[ece36606-bib-0003] Barve, A. , & Wagner, A. (2013). A latent capacity for evolutionary innovation through exaptation in metabolic systems. Nature, 500(7461), 203 10.1038/nature12301 23851393

[ece36606-bib-0004] Beltran, R. S. , Burns, J. M. , & Breed, G. A. (2018). Convergence of biannual moulting strategies across birds and mammals. Proceedings of the Royal Society B: Biological Sciences, 285(1878), 20180318 10.1098/rspb.2018.0318 PMC5966605 29769361

[ece36606-bib-0005] BirdLife International and Handbook of the Birds of the World (2016). Bird species distribution maps of the world. Version 6.0. Retrieved from http://datazone.birdlife.org/species/requestdis

[ece36606-bib-0006] Bivand, R. , & Lewin‐Koh, N. (2016). maptools: Tools for reading and handling spatial objects. R package version 0.8‐39. Retrieved from https://CRAN.R‐project.org/package=maptools

[ece36606-bib-0007] Bock, W. J. (1959). Preadaptation and multiple evolutionary pathways. Evolution, 13(2), 194–211.

[ece36606-bib-0008] Brunsdon, C. , & Chen, H. (2014). GISTools: Some further GIS capabilities for R. R package version 0.7‐4. Retrieved from https://CRAN.R‐project.org/package=GISTools

[ece36606-bib-0009] Butcher, G. S. , & Rohwer, S. (1989). The evolution of conspicuous and distinctive coloration for communication in birds In Power D.M. (Ed.). Current ornithology (pp. 51–108). Boston, MA: Springer.

[ece36606-bib-0010] Butler, M. A. , & King, A. A. (2004). Phylogenetic comparative analysis: A modeling approach for adaptive evolution. The American Naturalist, 164(6), 683–695. 10.1086/426002 29641928

[ece36606-bib-0011] Chadbourne, A. P. (1897). The spring plumage of the Bobolink, with remarks on ‘Color‐Change’ and ‘Moulting’. The Auk, 14(2), 137–149.

[ece36606-bib-0012] Chaine, A. S. , & Lyon, B. E. (2008). Adaptive plasticity in female mate choice dampens sexual selection on male ornaments in the lark bunting. Science, 319(5862), 459–462. 10.1126/science.1149167 18218896

[ece36606-bib-0013] Cheney, D. L. , & Seyfarth, R. M. (2005). Constraints and preadaptations in the earliest stages of language evolution. The Linguistic Review, 22(2–4), 135–159. 10.1515/tlir.2005.22.2-4.135

[ece36606-bib-0015] Curson, J. (2010). "New World warblers" Vol. 15. Weavers to New World Warblers: Family Parulide, Handbook of the birds of the world. Barcelona, Spain: Lynx Edicions. (Del Hoyo, Elliot, & Christie, eds).

[ece36606-bib-0016] Dale, J. , Dey, C. J. , Delhey, K. , Kempenaers, B. , & Valcu, M. (2015). The effects of life history and sexual selection on male and female plumage colouration. Nature, 527(7578), 367. 26536112 10.1038/nature15509

[ece36606-bib-0017] Darwin, C. (1981). The descent of man, and selection in relation to sex. 1871. Princeton, NJ: Princeton UP.

[ece36606-bib-0018] Dimond, C. C. , Cabin, R. J. , & Brooks, J. S. (2011). Feathers, dinosaurs, and behavioral cues: Defining the visual display hypothesis for the adaptive function of feathers in non‐avian theropods. Bios, 82(3), 58–64. 10.1893/011.082.0302

[ece36606-bib-0019] Dunn, J. , & Garrett, K. (1997). A field guide to warblers of North America. Boston, MA: Houghton Mifflin Harcourt.

[ece36606-bib-0020] Dunn, P. O. , Armenta, J. K. , & Whittingham, L. A. (2015). Natural and sexual selection act on different axes of variation in avian plumage color. Science Advances, 1(2), e1400155 10.1126/sciadv.1400155 26601146 PMC4643820

[ece36606-bib-0021] Friedman, N. R. , Hofmann, C. M. , Kondo, B. , & Omland, K. E. (2009). Correlated evolution of migration and sexual dichromatism in the New World orioles (Icterus). Evolution: International Journal of Organic Evolution, 63(12), 3269–3274. 19659597 10.1111/j.1558-5646.2009.00792.x

[ece36606-bib-0099] Froehlich, D. R. , Rohwer, S. , & Stutchbury, B. J. (2004). Pre‐breeding molt constraints versus winter territoriality. Is conspicuous winter coloration maladaptive? In Greenberg R. & Marra P. P. (Eds.). Birds of two worlds: the ecology and evolution of migration, (321–355). Baltimore, MD: Johns Hopkins University Press.

[ece36606-bib-0023] Garamszegi, L. Z. (Ed.) (2014). Modern phylogenetic comparative methods and their application in evolutionary biology: Concepts and practice. London, UK: Springer.

[ece36606-bib-0025] Gomez, D. , & Théry, M. (2004). Influence of ambient light on the evolution of colour signals: Comparative analysis of a Neotropical rainforest bird community. Ecology Letters, 7(4), 279–284. 10.1111/j.1461-0248.2004.00584.x

[ece36606-bib-0026] Götmark, F. , Post, P. , Olsson, J. , Himmelmann, D. , & Gotmark, F. (1997). Natural selection and sexual dimorphism: Sex‐biased sparrowhawk predation favours crypsis in female chaffinches. Oikos, 540–548. 10.2307/3546627

[ece36606-bib-0027] Hamilton, T. H. (1961). On the functions and causes of sexual dimorphism in breeding plumage characters of North American species of warblers and orioles. The American Naturalist, 95(881), 121–123. 10.1086/282167

[ece36606-bib-0028] Harmon, L. J. , Weir, J. T. , Brock, C. D. , Glor, R. E. , & Challenger, W. (2008). GEIGER: Investigating evolutionary radiations. Bioinformatics, 24, 129–131. 10.1093/bioinformatics/btm538 18006550

[ece36606-bib-0029] Hijmans, R. J. (2016). Raster: Geographic data analysis and modeling. R package version 2.5‐8. Retrieved from https://CRAN.R‐project.org/package=raster

[ece36606-bib-0030] Hijmans, R. J. , Cameron, S. , Parra, J. , Jones, P. G. , Jarvis, A. , & Richardson, K. (2005). Very high resolution interpolated climate surfaces for global land areas. International Journal of Climatology, 25(15), 1965–1978.

[ece36606-bib-0031] Hill, G. E. (1991). Plumage coloration is a sexually selected indicator of male quality. Nature, 350(6316), 337.

[ece36606-bib-0032] Holmgren, N. , & Hedenström, A. (1995). The scheduling of molt in migratory birds. Evolutionary Ecology, 9(4), 354–368. 10.1007/BF01237759

[ece36606-bib-0033] Howell, S. N. G. (2010). Molt in North American Birds. Boston, MA: Houghton Mifflin Harcourt.

[ece36606-bib-0035] Ito, S. , Wakamatsu, K. , & Sarna, T. (2018). Photodegradation of eumelanin and pheomelanin and its pathophysiological implications. Photochemistry and Photobiology, 94(3), 409–420. 10.1111/php.12837 28873228

[ece36606-bib-0036] Johnson, E. I. , Stouffer, P. C. , & Bierregaard, R. O. Jr (2012). The phenology of molting, breeding and their overlap in central Amazonian birds. Journal of Avian Biology, 43(2), 141–154. 10.1111/j.1600-048X.2011.05574.x

[ece36606-bib-0037] Johnson, E. I. , & Wolfe, J. D. (2017). Molt in Neotropical birds: Life history and aging criteria, Cleveland, OH: CRC Press.

[ece36606-bib-0038] Karubian, J. (2002). Costs and benefits of variable breeding plumage in the red‐backed fairy‐wren. Evolution, 56(8), 1673–1682. 12353760 10.1111/j.0014-3820.2002.tb01479.x

[ece36606-bib-0039] Ketola, T. , Mikonranta, L. , Zhang, J. I. , Saarinen, K. , Örmälä, A.‐M. , Friman, V.‐P. , … Laakso, J. (2013). Fluctuating temperature leads to evolution of thermal generalism and preadaptation to novel environments. Evolution, 67(10), 2936–2944. 10.1111/evo.12148 24094344

[ece36606-bib-0040] Kiat, Y. , Izhaki, I. , & Sapir, N. (2019). The effects of long‐distance migration on the evolution of moult strategies in Western‐Palearctic passerines. Biological Reviews, 94(2), 700–720. 10.1111/brv.12474 30334341

[ece36606-bib-0041] Lennox, F. G. , & Rowlands, R. J. (1969). Photochemical degradation of keratins. Photochemistry and Photobiology, 9(4), 359–367. 10.1111/j.1751-1097.1969.tb07300.x 5771419

[ece36606-bib-0042] Li, Q. , Gao, K.‐Q. , Meng, Q. , Clarke, J. A. , Shawkey, M. D. , D'Alba, L. , … Vinther, J. (2012). Reconstruction of Microraptor and the evolution of iridescent plumage. Science, 335(6073), 1215–1219. 22403389 10.1126/science.1213780

[ece36606-bib-0044] Lovette, I. J. , Pérez‐Emán, J. L. , Sullivan, J. P. , Banks, R. C. , Fiorentino, I. , Córdoba‐Córdoba, S. , … Bermingham, E. (2010). A comprehensive multilocus phylogeny for the wood‐warblers and a revised classification of the Parulidae (Aves). Molecular Phylogenetics and Evolution, 57(2), 753–770. 10.1016/j.ympev.2010.07.018 20696258

[ece36606-bib-0045] Lyon, B. E. , Eadie, J. M. , & Hamilton, L. D. (1994). Parental choice selects for ornamental plumage in American coot chicks. Nature, 371(6494), 240–243. 10.1038/371240a0

[ece36606-bib-0046] Lyon, B. E. , & Montgomerie, R. (2012). Sexual selection is a form of social selection. Philosophical Transactions of the Royal Society B: Biological Sciences, 367(1600), 2266–2273. 10.1098/rstb.2012.0012 PMC3391428 22777015

[ece36606-bib-0102] Marcondes, R. S. , & Brumfield, R. T. (2019). Fifty shades of brown: Macroevolution of plumage brightness in the Furnariida, a large clade of drab Neotropical passerines. Evolution, 73(4), 704–719. 30816993 10.1111/evo.13707

[ece36606-bib-0047] Martin, T. E. , & Badyaev, A. V. (1996). Sexual dichromatism in birds: Importance of nest predation and nest location for females versus males. Evolution, 50(6), 2454–2460. 28565684 10.1111/j.1558-5646.1996.tb03631.x

[ece36606-bib-0048] McQueen, A. , Kempenaers, B. , Dale, J. , Valcu, M. , Emery, Z. T. , Dey, C. J. , … Delhey, K. (2019). Evolutionary drivers of seasonal plumage colours: Colour change by moult correlates with sexual selection, predation risk and seasonality across passerines. Ecology Letters, 22(11), 1838–1849. 10.1111/ele.13375 31441210

[ece36606-bib-0049] Møller, A. P. , & Cuervo, J. J. (1998). Speciation and feather ornamentation in birds. Evolution, 52(3), 859–869. 28565248 10.1111/j.1558-5646.1998.tb03710.x

[ece36606-bib-0050] Mulder, R. A. , & Magrath, M. J. (1994). Timing of prenuptial molt as a sexually selected indicator of male quality in superb fairy‐wrens (*Malurus cyaneus*). Behavioral Ecology, 5(4), 393–400. 10.1093/beheco/5.4.393

[ece36606-bib-0051] Murphy, M. E. , King, J. R. , & Lu, J. (1988). Malnutrition during the postnuptial molt of white‐crowned sparrows: Feather growth and quality. Canadian Journal of Zoology, 66(6), 1403–1413. 10.1139/z88-206

[ece36606-bib-0053] Orme, D. , Freckleton, R. , Thomas, G. , Petzoldt, T. , Fritz, S. , Isaac, N. , & Pearse, W. (2013). caper: Comparative Analyses of Phylogenetics and Evolution in R. R Package Version 0.5.2. Retrieved from https://CRAN.R‐project.org/package=caper

[ece36606-bib-0054] Pagel, M. (1994). Detecting correlated evolution on phylogenies: A general method for the comparative analysis of discrete characters. Proceedings of the Royal Society of London. Series B: Biological Sciences, 255(1342), 37–45.

[ece36606-bib-0055] Paradis, E. , Claude, J. , & Strimmer, K. (2004). APE: Analyses of phylogenetics and evolution in R language. Bioinformatics, 20, 289–290. 14734327 10.1093/bioinformatics/btg412

[ece36606-bib-0056] Payne, R. B. (1984). Sexual selection, lek and arena behavior, and sexual size dimorphism in birds. Ornithological Monographs, 33, iii–52. 10.2307/40166729

[ece36606-bib-0057] Pearlstein, E. , Hughs, M. , Mazurek, J. , McGraw, K. , Pesme, C. , & Garcia‐Garibay, M. (2014, September). Correlations between photochemical damage and UV fluorescence of feathers In ICOM Committee for Conservation, ICOM‐CC: 17th Triennial Meeting, Melbourne, 15–19 September 2014: Preprints.

[ece36606-bib-0058] Persons, W. S. , & Currie, P. J. (2019). Feather evolution exemplifies sexually selected bridges across the adaptive landscape. Evolution, 73(9), 1686–1694. 10.1111/evo.13795 31359437

[ece36606-bib-0060] Pinheiro, J. , Bates, D. , DebRoy, S. , Sarkar, D. ; R Core Team (2016). Nlme: Linear and Nonlinear Mixed Effects Models_. R package version 3.1‐128. Retrieved from http://CRAN.R‐project.org/package=nlme

[ece36606-bib-0061] Poole, A. F. , Pyle, P. , Patten, M. A. , & Paulson, D. R. (2016). Black‐bellied Plover (*Pluvialis**squatarola*), version 3.0 In Rodewald P. G. (Ed.) The Birds of North America. Ithaca, NY: Cornell Lab of Ornithology https://birdsoftheworld.org/bow/species/bkbplo/cur/introduction?login

[ece36606-bib-0062] Prum, R. O. (2005). Evolution of the morphological innovations of feathers. Journal of Experimental Zoology Part B: Molecular and Developmental Evolution, 304(6), 570–579. 10.1002/jez.b.21073 16208685

[ece36606-bib-0063] Pyle, P. (1997a). Molt limits in North American passerines. North American Bird Bander, 22(2), 49–89.

[ece36606-bib-0064] Pyle, P. (1997b). Identification Guide to North American Birds Part I. Bolinas CA: Slate Creek Press.

[ece36606-bib-0101] Pyle, P (1997). Identification guide to North American birds: a compendium of information on identifying, ageing, and sexing "near‐passerines" and passerines in the hand. Bolinas CA: Slate Creek Press.

[ece36606-bib-0065] Pyle, P. (2009). Identification Guide to North American Birds Part II. Bolinas, CA: Slate Creek Press.

[ece36606-bib-0066] Pyle, P. , & Kayhart, R. (2010). Replacement of primaries during the prealternate molt of a Yellow Warbler. North American Bird Bander, 35(4), 178–181.

[ece36606-bib-0067] Quiñones, A. E. , & Pen, I. (2017). A unified model of Hymenopteran preadaptations that trigger the evolutionary transition to eusociality. Nature Communications, 8(1), 1–13. 10.1038/ncomms15920 PMC5490048 28643786

[ece36606-bib-0068] R Core Team (2016). R: A language and environment for statistical computing. Vienna, Austria: R Foundation for Statistical Computing Retrieved from https://www.R‐project.org/

[ece36606-bib-0069] Renfrew, R. B. , Frey, S. J. , & Klavins, J. (2011). Phenology and sequence of the complete prealternate molt of Bobolinks in South America. Journal of Field Ornithology, 82(1), 101–113. 10.1111/j.1557-9263.2010.00312.x

[ece36606-bib-0070] Reudink, M. W. , Studds, C. E. , Marra, P. P. , Kurt Kyser, T. , & Ratcliffe, L. M. (2009). Plumage brightness predicts non‐breeding season territory quality in a long‐distance migratory songbird, the American redstart *Setophaga ruticilla* . Journal of Avian Biology, 40(1), 34–41.

[ece36606-bib-0071] Revell, L. J. (2012). phytools: an R package for phylogenetic comparative biology (and other things). Methods in Ecology and Evolution, 3(2), 217–223. 10.1111/j.2041-210X.2011.00169.x

[ece36606-bib-0072] Rodewald, P. (Ed.) (2015). The Birds of North America. Ithaca, NY: Cornell Laboratory of Ornithology Retrieved from The Birds of North America: https://birdsna.org

[ece36606-bib-0073] Rohwer, S. , & Butcher, G. S. (1988). Winter versus summer explanations of delayed plumage maturation in temperate passerine birds. The American Naturalist, 131(4), 556–572. 10.1086/284806

[ece36606-bib-0074] Rubenstein, D. R. , & Lovette, I. J. (2009). Reproductive skew and selection on female ornamentation in social species. Nature, 462(7274), 786–789. 10.1038/nature08614 20010686

[ece36606-bib-0075] Sætre, G. , Dale, S. , & Slagsvold, T. (1994). Female pied flycatchers prefer brightly coloured males. Animal Behaviour, 48(6), 1407–1416. 10.1006/anbe.1994.1376

[ece36606-bib-0076] Schiestl, F. P. , & Cozzolino, S. (2008). Evolution of sexual mimicry in the orchid subtribe orchidinae: The role of preadaptations in the attraction of male bees as pollinators. BMC Evolutionary Biology, 8(1), 27 10.1186/1471-2148-8-27 18226206 PMC2267782

[ece36606-bib-0077] Schulenberg, T. S. (Ed.) (2019). Neotropical Birds Online. Ithaca, NY: Cornell Lab of Ornithology.

[ece36606-bib-0078] Seebacher, F. (2003). Dinosaur body temperatures: The occurrence of endothermy and ectothermy. Paleobiology, 29(1), 105–122. 10.1666/0094-8373(2003)029<0105:DBTTOO>2.0.CO;2

[ece36606-bib-0079] Shipley, B. (2016). Cause and correlation in biology: A user's guide to path analysis. Structural equations and causal inference with R. Cambridge, UK: Cambridge University Press.

[ece36606-bib-0080] Shultz, A. J. , & Burns, K. J. (2013). Plumage evolution in relation to light environment in a novel clade of Neotropical tanagers. Molecular Phylogenetics and Evolution, 66(1), 112–125. 10.1016/j.ympev.2012.09.011 23026808

[ece36606-bib-0081] Shutler, D. , & Weatherhead, P. J. (1990). Targets of sexual selection: Song and plumage of wood warblers. Evolution, 44(8), 1967–1977. 28564423 10.1111/j.1558-5646.1990.tb04303.x

[ece36606-bib-0082] Simpson, R. K. , Johnson, M. A. , & Murphy, T. G. (2015). Migration and the evolution of sexual dichromatism: Evolutionary loss of female coloration with migration among wood‐warblers. Proceedings of the Royal Society B: Biological Sciences, 282(1809), 20150375. 10.1098/rspb.2015.0375 PMC4590446 26019159

[ece36606-bib-0083] Slagsvold, T. , Dale, S. , & Kruszewicz, A. (1995). Predation favours cryptic coloration in breeding male pied flycatchers. Animal Behaviour, 50(4), 1109–1121. 10.1016/0003-3472(95)80110-3

[ece36606-bib-0052] Stackhouse, P.W. 2008 Surface Meteorology and Solar Energy (SSE) Data Release 6.0. http://eosweb.larc.nasa.gov/

[ece36606-bib-0084] Stephenson, T. , & Whittle, S. (2013). The Warbler Guide. Princeton, UK: Princeton University Press.

[ece36606-bib-0085] Stoddard, M. C. , & Prum, R. O. (2008). Evolution of avian plumage color in a tetrahedral color space: A phylogenetic analysis of new world buntings. The American Naturalist, 171(6), 755–776. 10.1086/587526 18419340

[ece36606-bib-0086] Stresemann, E. , & Stresemann, V. (1966). Die Mauser der Vögel. Journal of Ornithology, 107.

[ece36606-bib-0087] Surmacki, A. (2008). Preen waxes do not protect carotenoid plumage from bleaching by sunlight. Ibis, 150(2), 335–341. 10.1111/j.1474-919X.2007.00796.x

[ece36606-bib-0088] Swaddle, J. P. , Witter, M. S. , Cuthill, I. C. , Budden, A. , & McCowen, P. (1996). Plumage condition affects flight performance in common starlings: Implications for developmental homeostasis, abrasion and moult. Journal of Avian Biology, 27(2), 103–111. 10.2307/3677139

[ece36606-bib-0089] Terrill, R. S. (2018). Feather growth rate increases with latitude in four species of widespread resident Neotropical birds. The Auk: Ornithological Advances, 135(4), 1055–1063. 10.1642/AUK-17-176.1

[ece36606-bib-0090] Tökölyi, J. , Bókony, V. , & Barta, Z. (2008). Seasonal colour change by moult or by the abrasion of feather tips: A comparative study. Biological Journal of the Linnean Society, 94(4), 711–721. 10.1111/j.1095-8312.2008.01027.x

[ece36606-bib-0091] Underhill, L. G. , Prys‐Jones, R. P. , Dowsett, R. J. , Herroelen, P. , Johnson, D. N. , Lawn, M. R. , … Tree, A. J. (1992). The biannual primary moult of willow warblers *Phylloscopus trochilus* in Europe and Africa. Ibis, 134(3), 286–297. 10.1111/j.1474-919X.1992.tb03811.x

[ece36606-bib-0092] Von Hardenberg, A. , & Gonzalez‐Voyer, A. (2013). Disentangling evolutionary cause‐effect relationships with phylogenetic confirmatory path analysis. Evolution, 67(2), 378–387. 23356611 10.1111/j.1558-5646.2012.01790.x

[ece36606-bib-0093] Wallace, A. R. (1891). Natural selection and tropical nature: Essays on descriptive and theoretical biology. New York, NY: Macmillan and Company.

[ece36606-bib-0094] West‐Eberhard, M. J. (1979). Sexual selection, social competition, and evolution. Proceedings of the American Philosophical Society, 123(4), 222–234.

[ece36606-bib-0095] Whittow, G. C. , & Tazawa, H. (1991). The early development of thermoregulation in birds. Physiological Zoology, 64(6), 1371–1390. 10.1086/physzool.64.6.30158220

[ece36606-bib-0096] Winger, B. M. , Lovette, I. J. , & Winkler, D. W. (2011). Ancestry and evolution of seasonal migration in the Parulidae. Proceedings of the Royal Society B: Biological Sciences, 279(1728), 610–618. 10.1098/rspb.2011.1045 PMC3234563 21752818

[ece36606-bib-0097] Wolfe, J. (2011). Featured photo: First evidence for eccentric prealternate molt in the Indigo Bunting: Possible implications for adaptive molt strategies. Western Birds, 42(2011), 257–262.

[ece36606-bib-0098] Wolfe, J. D. , Johnson, E. I. , & Terrill, R. S. (2014). Searching for consensus in molt terminology 11 years after Howell et al'.s “first basic problem”. The Auk: Ornithological Advances, 131(3), 371–377.

